# Ventricular pro-arrhythmic phenotype, arrhythmic substrate, ageing and mitochondrial dysfunction in peroxisome proliferator activated receptor-γ coactivator-1β deficient (*Pgc-1β*^*−/−*^) murine hearts

**DOI:** 10.1016/j.mad.2018.05.004

**Published:** 2018-07

**Authors:** Shiraz Ahmad, Haseeb Valli, Karan R. Chadda, James Cranley, Kamalan Jeevaratnam, Christopher L.-H. Huang

**Affiliations:** a Physiological Laboratory, University of Cambridge, Downing Street, Cambridge, CB2 3EG, United Kingdom; b Faculty of Health and Medical Sciences, University of Surrey, GU2 7AL, Guildford, Surrey, United Kingdom; c PU-RCSI School of Medicine, Perdana University, 43400, Serdang, Selangor Darul Ehsan, Malaysia; d Department of Biochemistry, University of Cambridge, Tennis Court Road, Cambridge, CB2 1QW, United Kingdom

**Keywords:** Ventricular arrhythmia, Mitochondrial dysfunction, Fibrosis, Peroxisome proliferator activated receptor-γ coactivator-1 (PGC-1), Action potential conduction

## Abstract

**Introduction:**

Ageing and age-related bioenergetic conditions including obesity, diabetes mellitus and heart failure constitute clinical ventricular arrhythmic risk factors.

**Materials and methods:**

Pro-arrhythmic properties in electrocardiographic and intracellular recordings were compared in young and aged, peroxisome proliferator-activated receptor-γ coactivator-1β knockout (*Pgc-1β^−/−^*) and wild type (WT), Langendorff-perfused murine hearts, during regular and programmed stimulation (PES), comparing results by two-way ANOVA.

**Results and discussion:**

Young and aged *Pgc-1β^−/−^* showed higher frequencies and durations of arrhythmic episodes through wider PES coupling-interval ranges than WT. Both young and old, regularly-paced, *Pgc-1β^-/-^* hearts showed slowed maximum action potential (AP) upstrokes, (d*V*/d*t*)_max_ (∼157 *vs*. 120–130 V s^-1^), prolonged AP latencies (by ∼20%) and shortened refractory periods (∼58 *vs*. 51 ms) but similar AP durations (∼50 ms at 90% recovery) compared to WT. However, *Pgc-1β^-/-^* genotype and age each influenced extrasystolic AP latencies during PES. Young and aged WT ventricles displayed distinct, but *Pgc-1β^−/−^* ventricles displayed similar dependences of AP latency upon (d*V*/d*t*)_max_ resembling aged WT. They also independently increased myocardial fibrosis. AP wavelengths combining activation and recovery terms paralleled contrasting arrhythmic incidences in *Pgc-1β^-/-^* and WT hearts. Mitochondrial dysfunction thus causes pro-arrhythmic *Pgc-1β^−/−^* phenotypes by altering AP conduction through reducing (d*V*/d*t*)_max_ and causing age-dependent fibrotic change.

## Highlights

• Mitochondrial dysfunction occurs with both age and age-related chronic conditions.
• We modelled its ventricular arrhythmic effects using *Pgc-1β*^−/−^ murine hearts.
• *Pgc-1β*^*−/−*^ ventricles showed age-dependent pro-arrhythmic and fibrotic changes.
• These accompanied slowed action potential depolarisation and conduction.
• The latter could underly age- and metabolically-dependent ventricular arrhythmia.

## Graphical abstract

**Figure:**
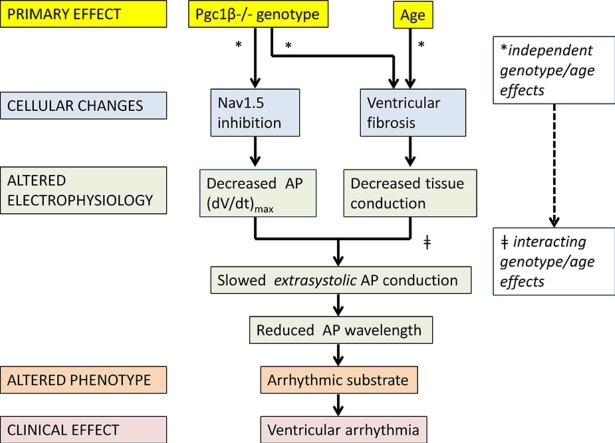

## 1 Introduction

Cardiovascular disease is the leading worldwide cause of mortality. Approximately half such cases are attributable to sudden cardiac death (SCD) (Go et al., 2013), often following ventricular arrhythmias. The latter follow disruption of the normally coordinated sequence of activation and inactivation of ion channel species underlying cardiac action potentials (AP). Models for several *monogenic* ion channel disorders using genetically-modified murine hearts have provided valuable insights into the contributions of particular channels to arrhythmic events (Huang, 2017). However, such conditions account for a relatively small proportion of SCDs in the clinical setting. Growing evidence also links such arrhythmias to energetic dysfunction seen in both ageing and age-related conditions including obesity, diabetes mellitus and heart failure (Hookana et al., 2011; Kucharska-Newton et al., 2010; Yeung et al., 2012). The latter constitute risk factors for SCD independent of any underlying coronary artery disease (Adabag et al., 2015; Yeung et al., 2012). Ageing itself is associated with an increased incidence of cardiac rhythm disturbances including both pathological bradycardic rhythms as well as atrial and ventricular tachy-arrhythmias (Bradshaw et al., 2014; Deo and Albert, 2012; Go et al., 2001), though the underlying mechanisms remain unclear.

*Biochemical consequences* of energetic deficiency have been studied in systems deficient in peroxisome proliferator activated receptor-γ coactivator-1 (PGC-1) transcriptional coactivators. These proteins regulate mitochondrial mass, function and cellular metabolism, upregulating expression of nuclear and mitochondrial genes involved in fatty acid β–oxidation, the tricarboxylic acid cycle and electron transport (Arany et al., 2005). In particular, PGC-1α and PGC-1β are highly expressed in oxidative tissues such as the heart, serving to co-ordinate mitochondrial activity with upstream cellular signals (Sonoda et al., 2007). They thus form a nexus for a range of metabolic pathways within the cardiomyocyte, central to the heart’s ability to meet energetic demands. Their expression is down-regulated in obesity, insulin resistance and type II diabetes mellitus along with an associated mitochondrial dysfunction (Dillon et al., 2012). Mice deficient in *both Pgc-1α* and *Pgc-1β* develop a perinatally lethal, low cardiac output state and conduction disease (Lai et al., 2008). In contrast, both *Pgc-1α^−/−^* and *Pgc*-1*β^−/−^* hearts show normal baseline function (Arany et al., 2005; Lelliott et al., 2006), with *Pgc*-1*β^−/−^* hearts displaying abnormal electrophysiological responses to adrenergic challenge. Together with its normal baseline contractile function these features make *Pgc-1β^−/−^* models attractive to investigating pro-arrhythmic effects of *chronic* mitochondrial dysfunction.

*Cellular electrophysiological abnormalities* have also been associated with energetic dysfunction. Firstly, an increased production of reactive oxygen species (ROS) affects maximum voltage-dependent Na^+^ and K^+^ current, Na^+^ and Ca^2+^ channel inactivation, late Na^+^ current (Liu et al., 2010; Wang et al., 2004), and ryanodine receptor and gap junction function (Brown and O’Rourke, 2010; Sovari et al., 2013; Terentyev et al., 2008). Secondly, ATP/ADP depletion accompanying mitochondrial dysfunction increases sarcolemmal ATP-sensitive K^+^ channel (sarcK_ATP_) open probabilities, shortening AP duration (Fischbach et al., 2004). Thirdly, oxidative stress and increased ROS formation may promote fibrotic change (Dai et al., 2009; Hafner et al., 2010), possibly through increased TGF-β activity (Brooks and Conrad, 2000; Rosenkranz et al., 2002), potentially disrupting gap junction function (Chilton et al., 2007; van Veen et al., 2005; Xie et al., 2009). Accordingly, studies in isolated *Pgc-1β^−/−^* cardiomyocytes specifically reported altered ion channel expression and function, abnormal Ca^2+^ homeostasis and delayed afterdepolarisation phenomena (Gurung et al., 2011).

These cellular changes have provided potential mechanisms altering cell-cell coupling (Smyth et al., 2010), AP conduction (Liu et al., 2010), repolarisation and refractoriness (Wang et al., 2004), and Ca^2+^-mediated triggering phenomena (Terentyev et al., 2008) in monogenic ion channel arrhythmic models. Structural abnormalities appearing as age-related fibrosis (Jeevaratnam et al., 2012, 2011), or compromised Na^+^ current activation through Nav1.5 deficiency proved pro-arrhythmic in *Scn5a*^+/−^ (Jeevaratnam et al., 2012, 2011; Martin et al., 2012) and *Scn5a*^+/ΔKPQ^ hearts (Wu et al., 2012) through altered AP activation and conduction. Similarly AP *recovery* abnormalities proved pro-aarrhythmic in *Scn5a*^+/Δkpq^ and *Kcne5*^-/-^ models for long QT syndromes (Sabir et al., 2008; Thomas et al., 2008, 2007). Altered intracellular Ca^2+^ homeostasis in *RyR2-P2328S* hearts both compromised Na^+^ currents (King et al., 2013b; Ning et al., 2016) and produced early or delayed afterdepolarization triggering events (Goddard et al., 2008; Hothi et al., 2008).

However, relatively few studies have investigated the electrophysiological consequences of these cellular changes and their implications for arrhythmic triggering or arrhythmic substrate at the level of *intact Pgc-1β^−/−^* hearts. Such hearts have shown potentially pro-arrhythmic APD alternans phenomena, and increased frequencies of ventricular tachycardia (VT) (Gurung et al., 2011; Lelliott et al., 2006), particularly with explorations through different steady-state pacing rates, the latter particularly in aged *Pgc-1β^−/−^* hearts. These were associated with reduced maximum action potential (AP) upstroke velocities, (d*V*/d*t*)_max_ and increased AP conduction latencies (Ahmad et al., 2017).

The present experiments characterise the electrophysiological mechanisms underlying arrhythmic substrates underlying these changes and how these progress with age in *Pgc-1β^−/−^* hearts modeling chronic mitochondrial dysfunction. They compared four groups of intact, young and aged, wild type (WT) and genetically modified, Langendorff-perfused *Pgc-1β^−/−^* hearts. Triggering events provoking arrhythmia in the presence of substrate were mimicked by S2 stimuli interposed at differing intervals following regular S1 pacing trains following protocols established on earlier occasions (Thomas et al., 2008, 2007). Direct intracellular determinations of resting membrane potentials (RMPs), AP amplitudes and latencies, and maximum rates of AP depolarisation, (d*V*/d*t*)_max,_ in cardiomyocytes *in situ* ensured unperturbed intracellular conditions, particularly of Ca^2+^ homeostasis. *Pgc-1β^-/-^* as opposed to WT genotypes were implicated in decreased (d*V*/d*t*)_max_ and increased AP latencies in the absence and in the presence of effects of age respectively. The latter segregation prompted explorations demonstrating distinct dependences of AP latency on (d*V*/d*t*)_max_ in young and aged WT hearts but a single such dependence in both *Pgc-1β^-/-^* groups approximating the functions observed in aged WT. The difference could be accounted for effects on AP latency of increases in fibrotic change arising from both *Pgc-1β^-/-^*genotype and ageing. Predictions of arrhythmic substrate from wavelengths derived from these AP activation and recovery terms, paralleled the relative incidences of arrhythmia in *Pgc-1β^−/−^* and WT hearts.

## 2 Materials & methods

### 2.1 Experimental animals

This research has been regulated under the Animals (Scientific Procedures) Act 1986 Amendment Regulations 2012 following ethical review by the University of Cambridge Animal Welfare and Ethical Review Body (AWERB). Age-matched homozygote *Pgc-1β^−/−^* and WT inbred C57/B6 mice were studied, with alternate male and female animals used in successive experiments within each group. *Pgc-1β^−/−^* mice were generated using a triple LoxP targeting vector as previously described (Lelliott et al., 2006). The young WT and young *Pgc-1β^−/−^* groups consisted of mice aged between 3–4 months; animals aged greater than 12 months were used for the aged WT and aged *Pgc-1β^−/−^* groups respectively. Mice were housed in plastic cages maintained at 21 ± 1 °C, subjected to 12 h dark/light cycles, and had unconstrained access to water, sterile rodent chow (RM3 Maintenance Diet, SDS, Witham, Essex, UK), bedding and environmental stimuli.

### 2.2 Whole heart Langendorff preparations

All chemical agents were purchased from Sigma-Aldrich (Poole, UK) except where otherwise indicated. Mice were first anticoagulated with 200 IU heparin sodium administered into the intra-peritoneal space with a 27 G hypodermic needle. After a 10 min interval, mice were killed by cervical dislocation (Schedule 1: UK Animals (Scientific Procedures) Act (1986)), a sternotomy performed, and the hearts were rapidly excised and submerged in ice-cold KH solution. The aorta was then cannulated with a modified 21 G hypodermic needle, secured with a 5-0 braided silk suture and retrogradely perfused with Krebs-Henseleit (KH) solution warmed to 37 °C by a water jacket heat-exchange coil (model C-85 A, Techne, Cambridge, UK) at a constant rate of 2.05 ml min^−1^ by a peristaltic pump (MINIPULS3, Gilson, Luton, UK) through 200 μm and 5 μm Millipore filters (Millipore, Watford, UK). The KH buffer was made with NaCl (119 mM), NaHCO_3_ (25 mM), KCl (4 mM), MgCl_2_ (1 mM), KH_2_PO_4_ (1.2 mM), CaCl_2_ (1.8 mM), glucose (10 mM) and sodium pyruvate (1.8 mM), bubbled with 95% O_2_/5% CO_2_ (British Oxygen Company, Manchester, UK) to achieve a pH of 7.4 preventing CaCO_3_ precipitation and matching the 7.3–7.4  pH of mouse plasma. Following commencement of perfusion, preparations were only further studied if they demonstrated sustained intrinsic activity with a basic cycle length (BCL) <200 ms and 1:1 atrioventricular conduction (AV) for 10 min. Hearts meeting these criteria were then perfused with 150 ml KH solution containing 20 μM blebbistatin after which perfusion with plain KH solution continued through to the conclusion of the experiment. The blebbistatin (20 μM, Selleckchem, Houston, USA) was used to electromechanically uncouple the heart during the microelectrode studies and permit stable cardiomyocyte impalement (Fedorov et al., 2007).

### 2.3 Electrophysiological recordings

Simultaneous microelectrode and electrocardiograph (ECG) studies were performed in a Faraday cage incorporating a modified horizontal Langendorff perfusion system, a light microscope (objective ×5, eyepiece ×5, W. Watson and Sons Limited, London, UK), custom-built head stage and a warmed tissue holding chamber. All equipment was electrically isolated. The stimulating and recording electrodes were positioned relative to the heart using two precision micromanipulators (Prior Scientific Instruments, Cambs, UK). Hearts were paced using a bipolar platinum-coated stimulating electrode (NuMed, New York, USA) positioned against the lateral surface of the right ventricle, connected to a DS2A-Mk.II stimulator (Digitimer, Welwyn Garden City, Herts., UK) delivering a voltage that was twice the diastolic excitation threshold plus 0.5 mV.

Whole heart volume conducted ECGs were recorded simultaneously with intracellular recordings to distinguish between local cellular and generalised organ level events. Two unipolar ECG leads were immersed into the warmed bath and placed adjacent to the right and left ventricles. Signals were amplified using model NL104 A amplifiers (NeuroLog; Digitimer, Hertfordshire, UK), filtered with model NL125/126 filters, set to a bandwidth of 5–500 Hz, and the analogue signal digitised using a model 1401 interface (Cambridge Electronic Design). Sharp microelectrodes for intracellular recordings were drawn from borosilicate glass pipettes (OD 1.2 mm, ID 0.69 mm, Harvard Apparatus, Cambridge, UK) using a homebuilt microelectrode puller, and filled with 3 M KCl (tip resistance 15–25 MΩ). The microelectrode was mounted onto a right-angled microelectrode holder containing a Ag/AgCl tip and connected to a high-input impedance direct-current microelectrode amplifier system (University of Cambridge, Cambridge, UK). Measurements of intracellular voltage were made relative to that of the Ag/AgCl reference electrode. An impalement was accepted for further recording if it resulted in an abrupt appearance of a resting membrane potential (RMP) between -65 mV and -90 mV, with APs that were normal in waveform and had an amplitude >75 mV. Impalements were restricted to the proximal left ventricle to avoid confounds of regional differences in AP characteristics. Hearts were first studied under conditions of regular pacing at a frequency of 8 Hz. This was followed by a programmed electrical stimulation (PES) protocol consisting of drive trains of eight paced (S1) beats at a BCL of 125 ms followed by a single extra stimulus (S2) every ninth beat. The initial S1-S2 coupling interval was 89 ms, reducing by 1 ms every subsequent cycle. The protocol was terminated when ventricular effective refractory period (ERP) was reached, defined as the final S1-S2 coupling interval at which the S2 stimulus successfully triggered an AP, or sustained arrhythmia was observed.

### 2.4 Quantification of AP parameters and arrhythmic incidence

AP amplitude was measured from the RMP to the peak voltage attained. The time from AP peak to repolarisation to 90% of baseline was taken as the action potential duration (APD_90_). We also obtained measures of APD_50_ and APD_70_ in order to provide fuller indications of AP recovery waveform. AP latencies were measured as the time intervening between the stimulus delivery and the AP peak. The maximum rate of depolarisation (d*V*/d*t*)_max_ was obtained from the first differential of the intracellular AP waveform. The incidence of abnormal rhythms was quantified from the PES protocol as follows: an isolated non-triggered AP following an S2 beat was termed an ectopic beat, and two successive non-triggered beats were termed a couplet. Episodes of ventricular tachycardia were categorised as non-sustained ventricular tachycardia (NSVT) if the episode consisted of ≥3 consecutive non-triggered beats but lasting <30 s; episodes lasting for >30 s were categorised as sustained VT.

### 2.5 Quantification of cardiac fibrosis

The degree of fibrotic change was assessed as previously described (Jeevaratnam et al., 2011). Briefly, following cardiectomy hearts were flushed with KH buffer, then perfused with 4% buffered formalin for 5 min, followed by storage in formalin overnight. After fixation, gross transverse sections of 7 μm thickness were cut and subjected to routine tissue processing, paraffin embedding and staining with picrosirius red. Sections were subsequently viewed, magnified and images digitally acquired using the Nano Zoomer 2.0 Digital Pathology system (Hamamatsu, Hertfordshire, UK). For quantification of fibrosis, a custom made 17 cm × 30 cm morphometric grid was superimposed on each photomicrograph, and each corresponding 0.2 mm × 0.2 mm area of tissue within the grid was assessed first for the presence or absence of cardiac tissue, and then for presence of fibrosis. The degree of fibrosis was expressed as the proportion of squares containing cardiac tissue that displayed evidence of fibrosis. The assessment was conducted independently by two investigators who were blinded to the animal identification, and their results were assessed for consistency by applying an inter-class correlation coefficient analysis (ICC), which can be interpreted as follows: 0–0.2 indicates poor agreement; 0.3 – 0.4 indicates fair agreement; 0.5 – 0.6 indicates moderate agreement; 0.7 – 0.8 indicates strong agreement; and >0.8 indicates almost perfect agreement.

### 2.6 Statistical analysis

The data was analysed with a custom-written programme using the python programming language and all statistical analysis performed in the R programming language (R Core Team, 2015). Data are expressed as means ± standard errors of the mean (SEM). Differences between experimental groups in AP parameters and degrees of fibrosis were compared using a two-way analysis of variance (ANOVA) testing for significant effects of genotype, ageing, and an interaction between the two. Where the *F*-ratio yielded a significant result, post-hoc Tukey honesty significant difference testing was performed. Categorical variables describing the incidence of arrhythmia were compared using a chi-squared test. Measures of arrhythmia duration included the number of beats during an episode of non-sustained (NSVT) or sustained ventricular tachycardia (VT), and the duration in seconds of the respective episode. Both were analysed with a multivariate negative binomial regression model. Risk factors and confounders were included in the multivariate analysis based on the regression analysis of each variable individually. Each variable could only be added to the multivariate model if it showed a significant effect in the simple univariate analysis. In all cases a p < 0.05 was taken to be significant, with application of Bonferroni correction where appropriate.

## 3 Results

The experiments made simultaneous ECG recordings in intact hearts and intracellular microelectrode recordings from ventricular cardiomyocytes *in situ*. The intracellular recordings employed microelectrode impalement sites confined to the proximal region of the left ventricle and consistent stimulating electrode positioning between hearts, minimising variabilities in distance between stimulating and recording electrode sites. They explored and characterised effects of the *Pgc-1β^−/−^* genotype and ageing upon arrhythmic properties at the organ level in Langendorff-perfused murine hearts during both regular pacing and programmed electrical stimulation (PES). These findings were then correlated with cellular electrophysiological quantifications of action potential (AP) activation and propagation, as well as recovery characteristics, and morphometric assessments of age-related structural change, features previously implicated in arrhythmic substrate.

The intracellular recordings confirmed fully polarised resting membrane potentials (RMPs) in all groups studied (young WT (n = 27): -79.24 ± 1.34 mV; aged WT (n = 27): -78.99 ± 1.30 mV; young *Pgc-1β^−/^* (n = 37)*-:* -74.76 ± 0.54 mV; aged *Pgc-1β^-/-^* (n = 29): -79.76 ± 1.40 mV) with slightly more polarised RMPs in young *Pgc-1β^-/-^* compared to each of the remaining groups (p < 0.05 in each case). AP amplitudes (young WT (n = 27): 82.89 ± 0.90 mV; aged WT (n = 27): 88.09 ± 1.3 mV; young *Pgc-1β^−/^* (n = 37)*-:* 79.57 ± 1.29 mV; aged *Pgc-1β^-/-^* (n = 29): 80.91 ± 1.19 mV), were slightly greater in aged WT than in each of the remaining experimental groups (p < 0.05 in each case). Nevertheless, comparisons of RMPs and AP amplitudes confirmed AP overshoots were positive as expected from recordings from viable ventricular cardiomyocytes.

### 3.1 *Pgc-1β*^-/-^ ventricles develop arrhythmic phenotypes

**Fig. 1:**
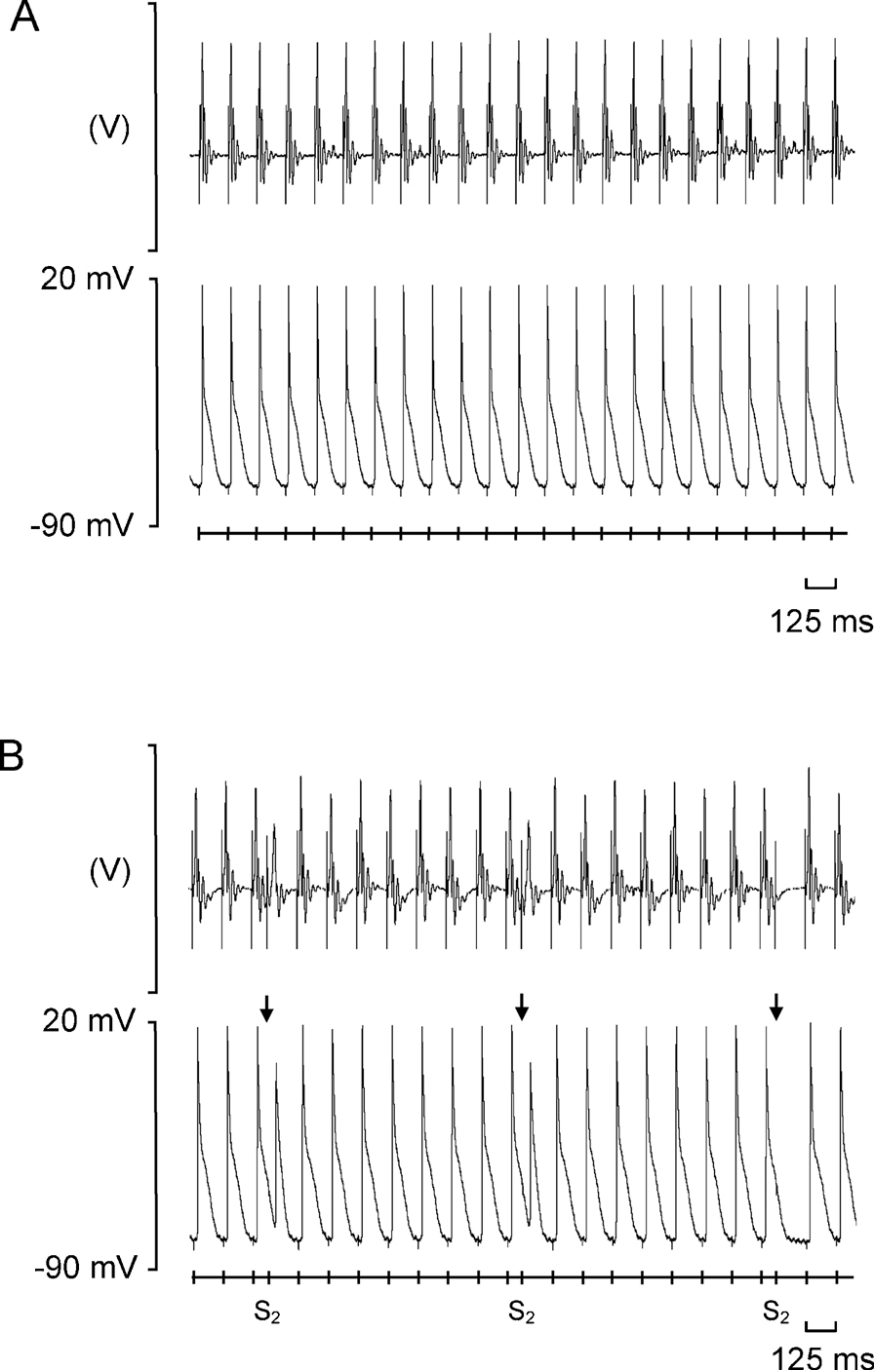
Typical electrocardiographic (ECG) (upper panel in each) and simultaneous intracellular action potential (AP) recordings (lower panel in each) obtained during 8 Hz pacing (A) and programmed electrical stimulation (PES) (B), recorded from a young wild type (WT) heart. The timings of stimulus delivery are given in the bar beneath the AP recordings, and corresponding stimulation artefacts can be seen on the ECG and AP traces, preceding the respective complexes. In panel (B), arrows indicate the imposition of S2 extrastimuli. The first two S2 stimuli trigger APs, whereas the third S2 stimulus fails to elicit a response, thus representing a refractory outcome.

The occurrence of *spontaneous* arrhythmic events were first quantified during regular 8 Hz pacing resembling normal resting heart rates. *Arrhythmic substrate* was thereafter assessed by applying extrasystolic S2 stimuli in a programmed electrical stimulation (PES) protocol. Fig. 1 shows typical ECG (upper trace in each panel) and intracellular AP recordings (lower trace in each panel) obtained during such regular 8 Hz pacing (A) and PES (B) from a young WT ventricle. The PES protocol comprised cycles of eight S1 beats at a baseline BCL of 125 ms followed by an extra stimulus (S2) every ninth beat. The initial S1-S2 coupling interval was 89 ms, decrementing by 1 ms with each cycle. Differences in arrhythmic propensity were quantified from occurrences or otherwise of both spontaneous and provoked arrhythmic events, and the S1-S2 intervals at which the latter took place.

**Fig. 2:**
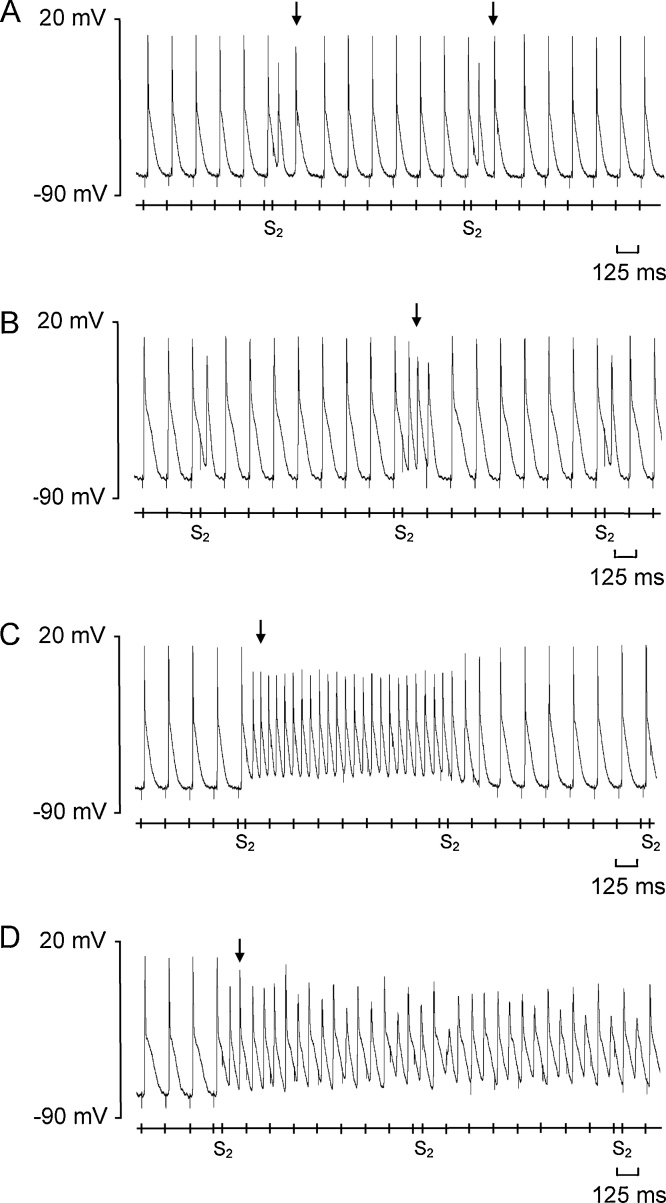
Typical AP recordings of different abnormal rhythms observed during programmed electrical stimulation (PES) in an aged *Pgc-1β^−/−^* heart following an extrasystolic S2 stimulus. These were classified as an individual ectopic beat (A), a couplet of two successive ectopic beats (B), non-sustained ventricular tachycardia (NSVT) (C) and sustained ventricular tachycardia (D). The timings of stimulus delivery are given in the bar beneath the AP recordings and arrows indicate the onset of the abnormal rhythm.

**Fig. 3:**
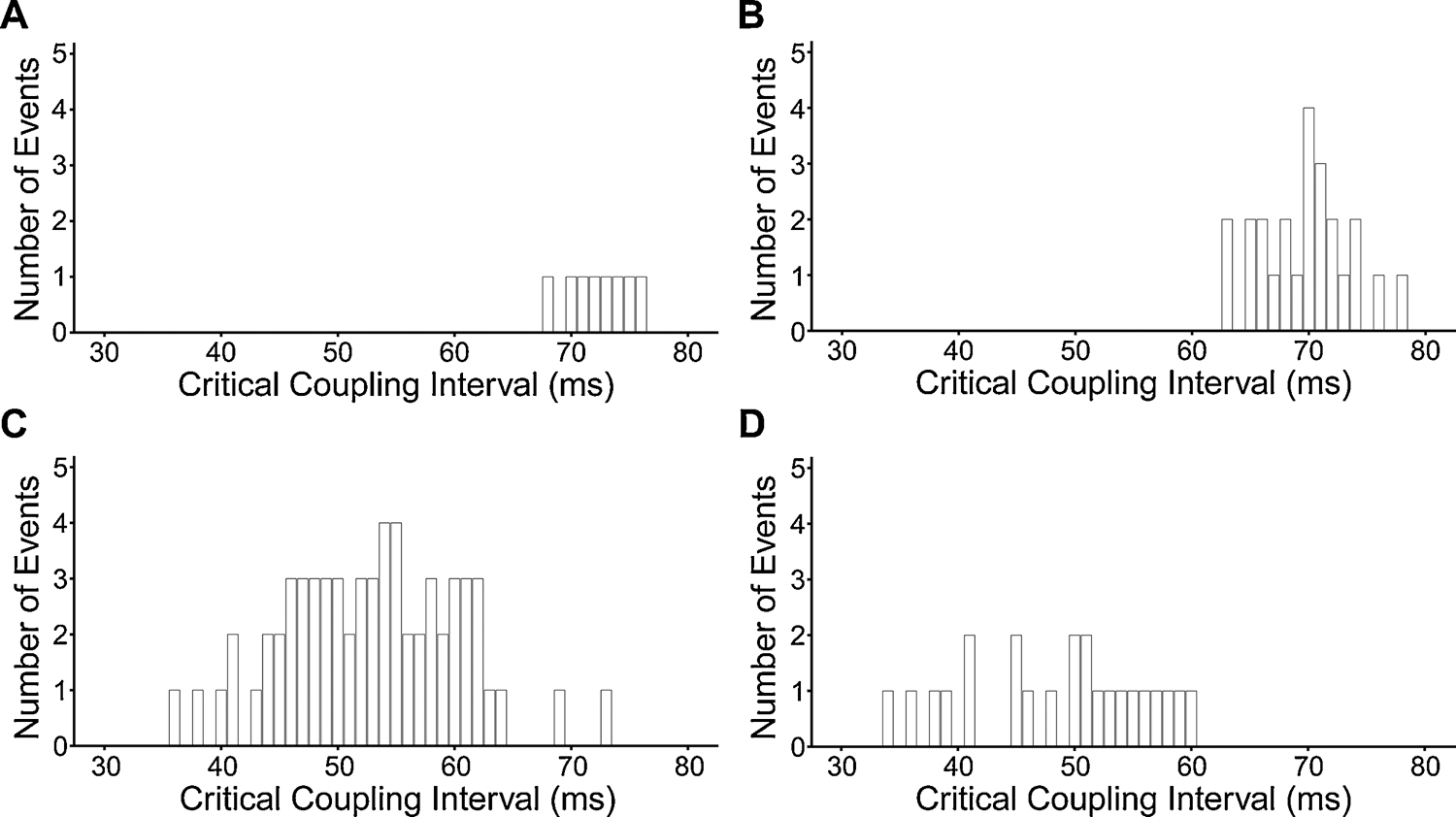
Occurrences of episodes of non-sustained ventricular tachycardia or sustained tachycardia recording during PES protocols in (A) young WT, (B) aged WT, (C) young *Pgc-1β^−/−^* and (D) aged *Pgc-1β^−/−^* hearts, stratified according to the S1–S2 coupling interval (critical coupling interval) that provoked the arrhythmia.

None of the hearts in any of the experimental groups showed spontaneous arrhythmic events during the regular 8 Hz pacing. In contrast, the PES protocols elicited a range of abnormal rhythms, exemplified in Fig. 2 for an aged *Pgc-1β^−/−^* ventricle. These included single (A) or pairs (couplets) of successive ectopic beats (B), non-sustained (C) and sustained ventricular tachycardia (D). Table 1 summarises frequencies with which these different categories of abnormal rhythms occurred stratified by experimental group. Young *Pgc-1β^−/−^* ventricles demonstrated greater incidences of rhythm disturbances than other experimental groups, particularly in the form of non-sustained ventricular tachycardia (NSVT) (p < 0.001). Young WT ventricles demonstrated few abnormal rhythms of any description; all the NSVT episodes observed occurred in the same heart. Aged *Pgc-1β^−/−^* displayed fewer individual episodes of abnormal rhythms than young *Pgc-1β^−/−^* hearts, and a similar number as aged WT hearts. The durations of the VT episodes, whether non-sustained or sustained, were influenced by interacting effects of age and genotype with the VT episodes in aged *Pgc-1β^−/−^* hearts significantly longer in duration than in the other groups (p < 0.05). Aged *Pgc-1β^−/−^* hearts were also the only experimental group showing sustained VT episodes. Thus an S2 beat could trigger an episode of a short and self-terminating run of NSVT in a young *Pgc-1β^−/−^* heart, with subsequent S2 beats triggering further short-lived episodes. In contrast, an episode of VT in an aged *Pgc-1β^−/−^* ventricle was more likely to be prolonged thereby precluding subsequent S2 beats from triggering further episodes. Finally, Fig. 3 compares incidences of NSVT and sustained VT in young (A, C) and aged (B, D) WT (A, B) and *Pgc-1β^−/−^* hearts (C, D), sorted by the S1S2 coupling interval at which the episodes took place. Both young and aged *Pgc-1β^−/−^* hearts showed arrhythmic phenomena over more extensive ranges of S1-S2 coupling intervals than the young and aged WT hearts.

**Table 1:** Summary of arrhythmic events according to experimental group.

Experimental Group	Numbers that developed NSVT or VT	Ectopic Beats	Couplets	NSVT	Sustained VT	Duration of VT Episode	
	(n / total)	Mean (±SEM)	Mean (±SEM)	Mean (±SEM)	Mean (±SEM)	No. of Beats (mean ± SEM)	Time (s) (mean ± SEM)
Young Wild Type	1 / 31	0	0.03 (±0.03)	0.26 (±0.26)	0	4.13 ± 0.74	0.022 ± 0.004
Aged Wild Type	4 / 29	0	0.03 (±0.03)	0.89 (±0.47)	0	5.96 ± 0.66	0.032 ± 0.004
Young *Pgc-1β^−/−^*	7 / 37	0.09 (±0.04)	1 (±0.48)	1.70 (±0.80)*	0	6.81 ± 2.00	0.041 ± 0.016
Aged *Pgc-1β^−/−^*	6 / 29	0.14 (±0.10)	0.10 (±0.30)	0.72 (±0.43)	0.07 (±0.05)	103.57 ± 64.32*	7.835 ± 3.765*

Symbols denote significant difference of value from corresponding measurements in the other experimental groups based on post hoc analysis, performed if the *F* value from two-way ANOVA was significant. Single, double and triple symbols denote p < 0.05, p < 0.01 and p < 0.001 respectively.

### 3.2 Altered AP parameters in *Pgc-*1β*^-/^*^-^ ventricles during regular pacing

The intracellular cardiomyocyte recordings next correlated the above arrhythmic phenotypes in the different groups with their corresponding electrophysiological parameters describing APs resulting from the regular 8 Hz pacing (Table 2). *Pgc-1β* ablation independently altered *both* AP activation and recovery properties in directions compatible with pro-arrhythmic defects in an *absence* of effects of ageing whether independently, or interacting with genotype. Thus, *maximum AP upstroke velocities*, (d*V*/d*t*)_max_, were reduced in *Pgc-1β^−/−^* compared to WT ventricles (F = 31.606, p < 0.001), without effects of age (F = 1.973, p > 0.05) or interacting effects of age and genotype (F = 0.904, p > 0.05). Post hoc tests confirmed lower (d*V*/d*t*)_max_ in both young and aged, *Pgc-1β^−/−^* group than either WT group. Assessments of AP conduction similarly demonstrated longer AP latencies between stimulus delivery and the AP peak, in *Pgc-1β^−/−^* than WT ventricles (F = 11.458, p < 0.001) without effects of age (F = 0.494, p > 0.05) or interactions between age and genotype (F = 0.744, p > 0.05). However, post hoc analysis demonstrated shorter AP latencies in young WT than both young (p < 0.05) and aged *Pgc-1β^−/−^* (p < 0.05) ventricles, but no differences between aged WT ventricles and the remaining groups.

**Table 2:** Action potential properties in WT and *Pgc-1β*^-/-^ hearts during regular 8 Hz pacing.

Experimental Group (n)	(d*V*/d*t*)_max_ (V s^−1^)	AP latency (ms)	APD_90_ Duration (ms)	APD_70_ Duration (ms)	APD_50_ Duration (ms)	Effective Refractory Period (ms)
Young Wild Type (27)	156.08 (±6.00)^***, †^	9.70 (±0.32)^*, †^	51.01 (±1.47)	27.51 (±1.29)	7.27 (±0.64)	57.15 (±1.92)
Aged Wild Type (27)	158.14 (±5.45)^‡‡‡, α^	10.92 (±0.35)	51.24 (±1.68)	26.08 (±1.57)	7.63 (±0.63)	59.92 (±2.20)^*, †^
Young *Pgc-1β^−/−^* (37)	119.23 (±5.49)^***, ‡‡‡^	12.85 (±0.77)^*^	47.31 (±2.12)	23.58 (±1.67)	8.69 (±1.00)	51.54 (±1.74)^*^
Aged *Pgc-1β^−/−^* (29)	132.21 (±5.73) ^†, α^	12.79 (±1.09) ^†^	53.14 (±1.60)	28.19 (±1.47)	8.27 (±0.54)	51.25 (±2.42) ^†^

All values are given as mean (±SEM).

Symbols denote significant difference on post hoc analysis between pairs of experimental groups, performed if the *F* value from two-way.

ANOVA was significant. Single, double and triple symbols denote p < 0.05, p < 0.01 and p < 0.001 respectively.

Of recovery characteristics, AP durations at 90%, 70% and 50% repolarisation, APD_90_, APD_70_ and APD_50_ respectively, were indistinguishable between groups. Finally, ventricular effective refractory periods (ERPs) were evaluated from the PES protocol as the shortest S1-S2 coupling interval at which an S2 stimulus successfully triggered a ventricular beat. *Pgc-1β^−/−^* ventricles showed shorter ERPs than WT (F = 13.508, p < 0.001) without effects of either age (F = 0.208, p > 0.05) or interactions between age and genotype (F = 0.391, p > 0.05). Post hoc Tukey tests demonstrated shorter ERPs in both young (p < 0.05) and aged *Pgc-1β^−/−^* (p < 0.05) compared to aged WT ventricles.

### 3.3 Altered AP parameters in *Pgc-1β*^*-/-*^ ventricles subjected to extrasystolic stimuli

**Fig. 4:**
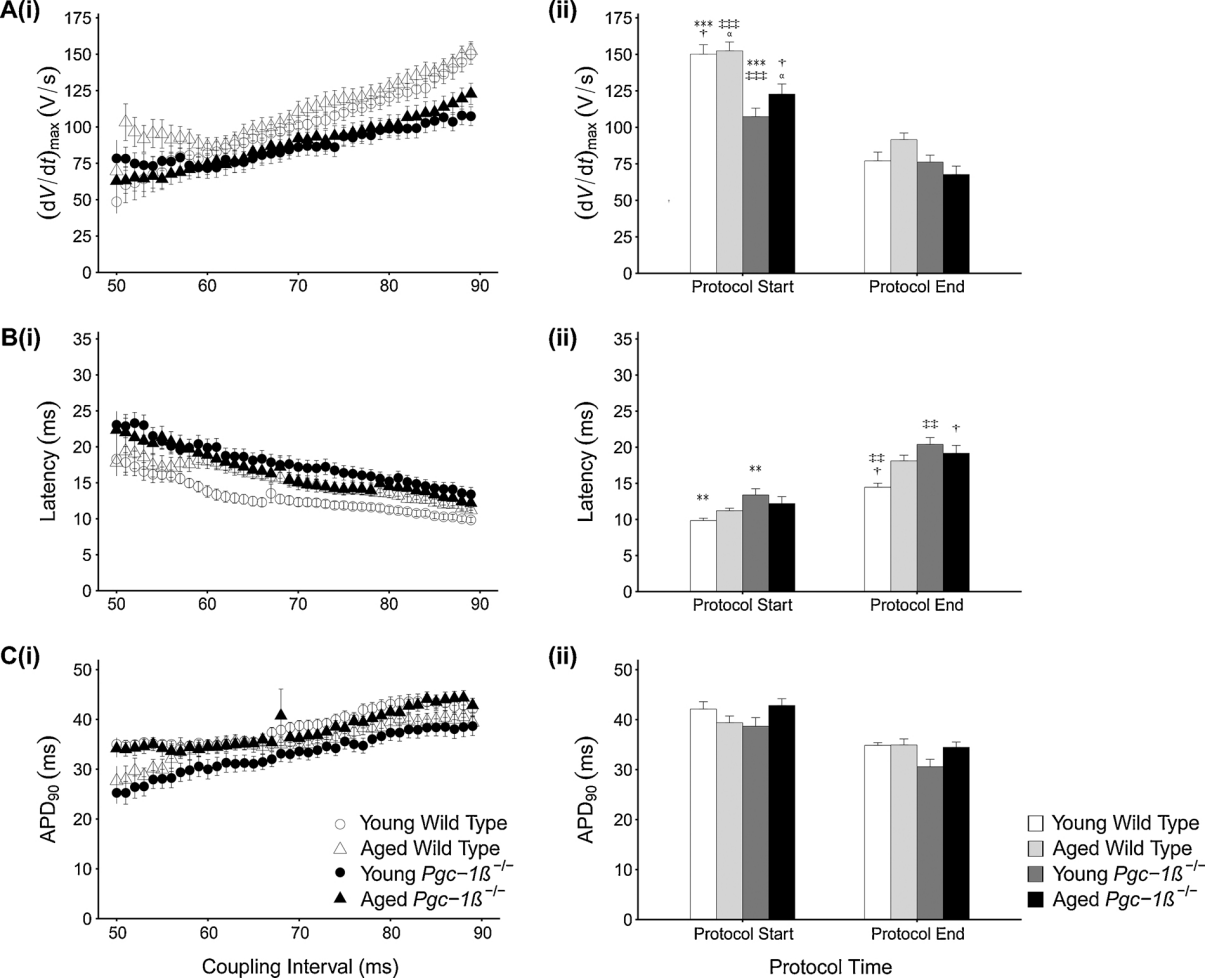
Means ± SEM of maximum action potential upstroke rate, (d*V*/d*t*)_max_ (A), AP latencies (B) and action potential durations to 90% recovery, APD_90_ (C) for S2 triggered APs in the four experimental groups for (i) S1–S2 intervals falling from 89 ms to 50 ms during the PES protocols. Panel (ii) for each provides a comparison of these values at the outset and at the end of the pacing protocol, corresponding to a refractory outcome or the onset of sustained arrhythmia. The symbols denote significant differences between each pair, obtained from post hoc Tukey testing, which was conducted if the ANOVA indicated a significant outcome. Single, double and triple symbols denote p < 0.05, p < 0.01 and p < 0.001 respectively.

PES protocols exploring for arrhythmic substrate in response to provocation by S2 extra stimuli gave ectopic APs whose variations of (d*V*/d*t*)_max_ and of AP latency with S1-S2 coupling intervals showed contrasting dependences upon genotype and age, despite invariant dependences of APD_90_ upon coupling interval. Thus, plots of (d*V*/d*t*)_max_ against S1-S2 coupling interval (Fig. 4A(i)) confirmed lower (d*V*/d*t*)_max_ values in *Pgc-1β^−/−^* compared to WT ventricles (F = 30.167, p < 0.001) without effects of age or interactions of age and genotype, at the long coupling intervals early in the PES protocol, in keeping with findings from the regular 8 Hz pacing. Post hoc testing demonstrated lower (d*V*/d*t*)_max_ in each individual *Pgc-1β^−/−^* group compared to any WT group (Fig. 4A(ii)), but no differences between young and aged ventricles within either group. (d*V*/d*t*)_max_ fell with shortening S1-S2 coupling interval preserving their relative magnitudes between experimental groups consistent with progressive reductions in conduction velocity and the increased arrhythmic tendency. The (d*V*/d*t*)_max_ values then converged to indistinguishable values whether stratified by genotype (F = 1.395, p > 0.05) or age (F = 0.060, p > 0.05) (Fig. 4A(ii)).

The corresponding AP latencies (Fig. 4B(i)) were longer in *Pgc-1β^−/−^* than WT ventricles at the longest S1-S2 coupling intervals (F = 8.633, p < 0.01) without independent effects of age (F = 0.001, p > 0.05) or interacting effects of age and genotype (F = 2.689, p > 0.05), consistent with the (d*V*/d*t*)_max_ readings. However, young *Pgc-1β^−/−^* showed longer AP latencies than young WT ventricles on post hoc Tukey analysis (p < 0.05) though with no further differences. Furthermore, AP latencies increased with falling S1-S2 intervals to *varying* extents amongst groups. AP latencies at the shortest S1-S2 intervals were affected by interactions between genotype and age (F = 4.100, p < 0.05). Young WT ventricles here showed shorter AP latencies than the remaining groups (post hoc Tukey tests: young WT *vs.* young *Pgc-1β^−/−^* p < 0.01; young WT *vs.* aged *Pgc-1β^−/−^*: p < 0.05; no significant differences in the remaining comparisons). AP latency did not differ between aged WT and either young or aged *Pgc-1β^−/−^* ventricles. Thus at the shortest S1-S2 coupling intervals, the young WT ventricles, which were the least arrhythmic, showed smaller increases in AP latency than any other group including aged WT ventricles.

Finally, similar shortenings of APD_90_ with reductions in S1-S2 coupling intervals (Fig. 4C(i)) were observed through all groups, with indistinguishable APD_90_ at both the longest (genotype: F = 0.004, p > 0.05; age: F = 0.309, p > 0.05) and shortest S1-S2 intervals (genotype: F = 2.661, p > 0.05; age: F = 2.152, p > 0.05) (Fig. 4C(ii)). Table 3 demonstrates that these similarities in action potential recovery extended to the respective values of APD_50_ and APD_70_ at the longest and shortest S1-S2 intervals.

**Table 3:** Action potential duration time in WT and *Pgc-1β^−/−^* hearts during programmed stimulation.

Experimental Group (n)	APD_50_ Duration (ms) Protocol Start	APD_50_ Duration (ms) Protocol End	APD_70_ Duration (ms) Protocol Start	APD_70_ Duration (ms) Protocol End	APD_90_ Duration (ms) Protocol Start	APD_90_ Duration (ms) Protocol End
Young Wild Type (27)	6.51 (±0.50)	9.98 (±0.63)	22.56 (±1.03)	20.63 (±0.50)	42.11 (±1.48)	34.86 (±0.54)
Aged Wild Type (27)	6.78 (±0.58)	8.98 (±0.75)	20.00 (±1.13)	20.42 (±1.02)	39.38 (±1.33)	34.95 (±1.19)
Young *Pgc-1β^−/−^* (37)	8.73 (±0.92)	8.15 (±0.86)	20.19 (±1.30)	16.93 (±1.20)	38.67 (±1.73)	30.60 (±1.48)
Aged *Pgc-1β^−/−^* (29)	7.74 (±0.61)	9.12 (±0.61)	22.85 (±0.99)	19.69 (±0.75)	42.84 (±1.34)	34.46 (±1.06)

All values are given as mean (±SEM).

Symbols denote significant difference on post hoc analysis between pairs of experimental groups, performed if the *F* value from two-way ANOVA was significant.

Single, double and triple symbols denote p < 0.05, p < 0.01 and p < 0.001 respectively.

### 3.4 Distinct dependences of AP latency upon (d*V*/d*t*)_max_ in WT and **Pgc-1β*^-/-^* ventricles

**Fig. 5:**
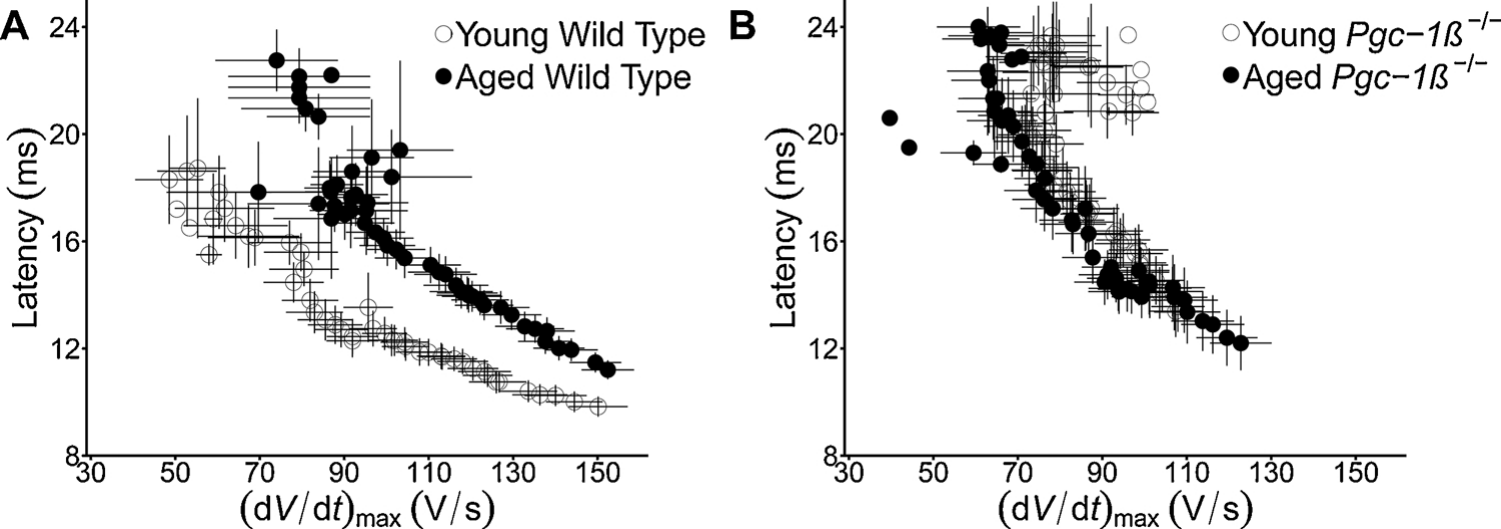
Dependences of AP latency upon (d*V*/d*t*)_max_ through the programmed electrical stimulation (PES) protocol compared in (A) young and old WT and (B) young and aged *Pgc-1β^−/−^* hearts.

These detailed differences in the effects of genotype and age upon the dependences of AP latency and *(*d*V/*d*t)*_max_ upon S1-S2 coupling interval prompted investigations of the relationship between these two parameters. Fig. 5 plots mean (±SEM) latencies of the extrasystolic S2 APs against their corresponding (d*V*/d*t*)_max_ values for each experimental group. Both WT and *Pgc-1β^−/^*^-^ showed AP latencies increasing with falling (d*V*/d*t*)_max_ and shortening S1-S2 coupling intervals, consistent with much of this increased AP latency being related to falls in (d*V*/d*t*)_max_. However, young and aged WT ventricles generated two separable plots of AP latency against (d*V*/d*t*)_max_ (Fig. 5A), with aged showed greater AP latencies than young WT ventricles at any given (d*V*/d*t*)_max_. In contrast, *both* young and aged *Pgc-1β^-/-^* ventricles gave similar AP latency - (d*V*/d*t*)_max_ plots (Fig. 5B), whose values closely resembled those of *aged* as opposed to young WT.

### 3.5 Increased fibrotic change with *Pgc-1β* ablation

**Fig. 6:**
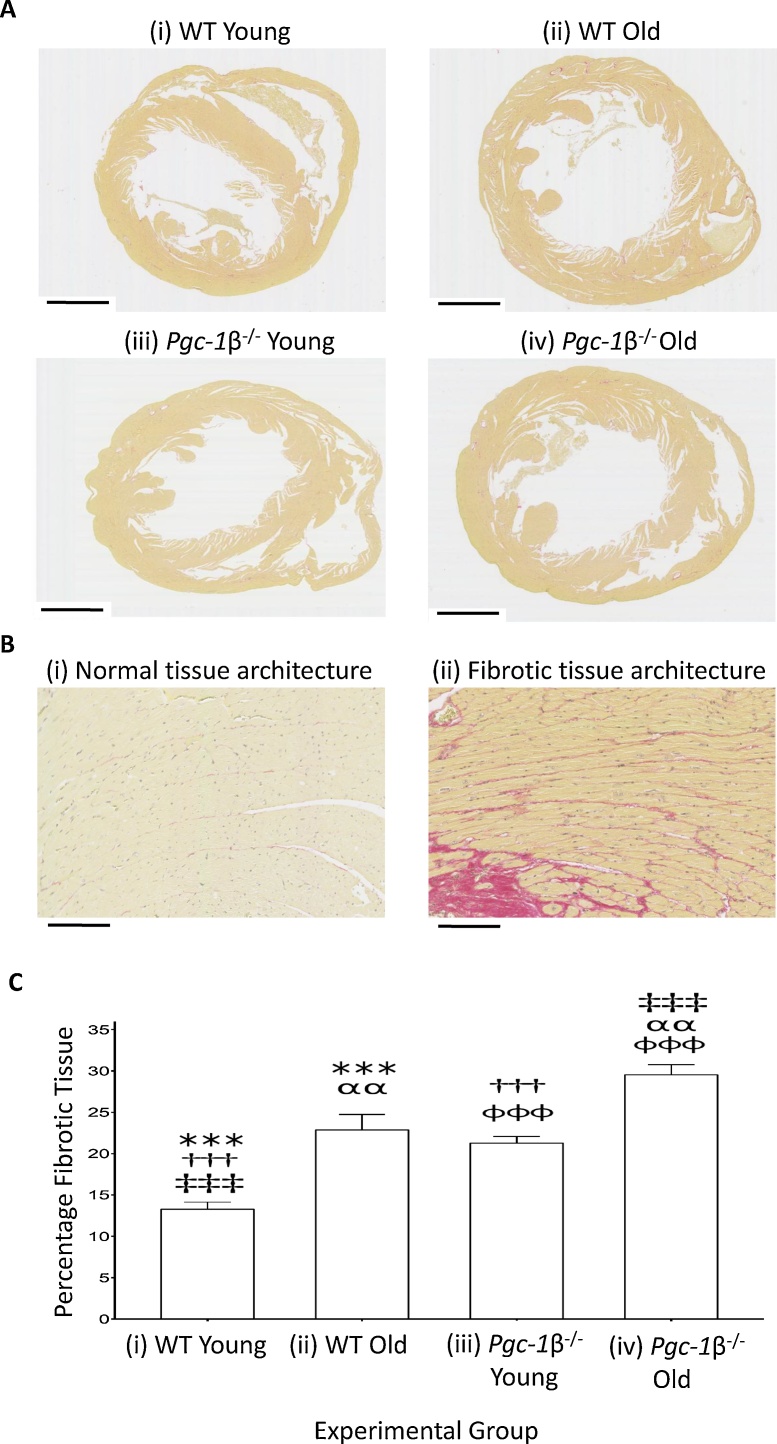
(A) Representative examples of histological samples from young ((i) and (iiii)) and old ((ii) and (iv)), WT ((i) and (ii)) and *Pgc1β*^−/−^ hearts ((iii) and (iv)) used for morphological assessment of fibrotic change (scale bar 1 mm), with (B) typical higher power appearances to illustrate (i) normal tissue architecture in young WT and (ii) tissue structure showing fibrotic change in aged *Pgc-1β^-/-^* ventricles (scale bar 100 μm). (C) The degree of fibrotic change was assessed as the proportion of morphometric squares covering tissue that showed positive evidence of fibrotic change as detected by picrosirius red staining. Numbers of hearts examined: young WT (n = 8), aged WT (n = 8), young *Pgc-1β^-/-^* (n = 9), aged *Pgc-1β^-/-^* (n = 10). Symbols denote pairs of points showing significant differences from post hoc Tukey testing, where single, double and triple symbols denote p < 0.05, p < 0.01 and p < 0.001 respectively (For interpretation of the references to colour in this figure legend, the reader is referred to the web version of this article).

Young and aged WT ventricles thus showed distinct dependences of AP latency upon (d*V*/d*t*)_max_ whereas both young and aged *Pgc-1β^−/−^* ventricles showed a single dependence resembling that shown by the aged as opposed to the young WT. (d*V*/d*t*)_max_ classically reflects cardiomyocyte membrane depolarisation driven by regenerative inward Na^+^ current important in AP conduction (King et al., 2013c; Zhang et al., 2013). However, AP conduction additionally varies with tissue conductivity properties reflecting gap junction resistances separating successive coupled cardiomyocytes, and their cell membrane capacitances influenced by fibroblast-myocyte fusion (Davies et al., 2014; King et al., 2013a). Previous reports in murine *Scn5a^+/−^* hearts had implicated age-dependent fibrotic change and such effects on tissue conductivity in similar pro-arrhythmic alterations in AP conduction (Jeevaratnam et al., 2012, 2011; Royer et al., 2005). The final experiments accordingly made morphometric assessments for fibrotic change amongst the four experimental groups. This was conducted blindly by two independent investigators achieving a high measure of consistency (ICC = 0.94). Fig. 6 illustrates typical histological sections from young and aged WT and *Pgc-1β^-/-^* hearts (A), higher power representations of normal and fibrotic tissue (B) and quantifications of this fibrotic change (C). Age and genotype independently increased levels of fibrosis in ventricular tissue (p < 0.05 for both), with no evidence of interaction between variables. The differing fibrotic levels in aged *vs*. young WT ventricles paralleled their differing AP latency - (d*V*/d*t*)_max_ association, whereas the similar fibrotic levels in young *Pgc-1β^-/-^* and aged WT ventricles paralleled their similar AP latency - (d*V*/d*t*)_max_ plots.

### 3.6 Action potential wavelengths and pro-arrhythmic phenotypes in *Pgc-1β^-/-^* ventricles

**Fig. 7:**
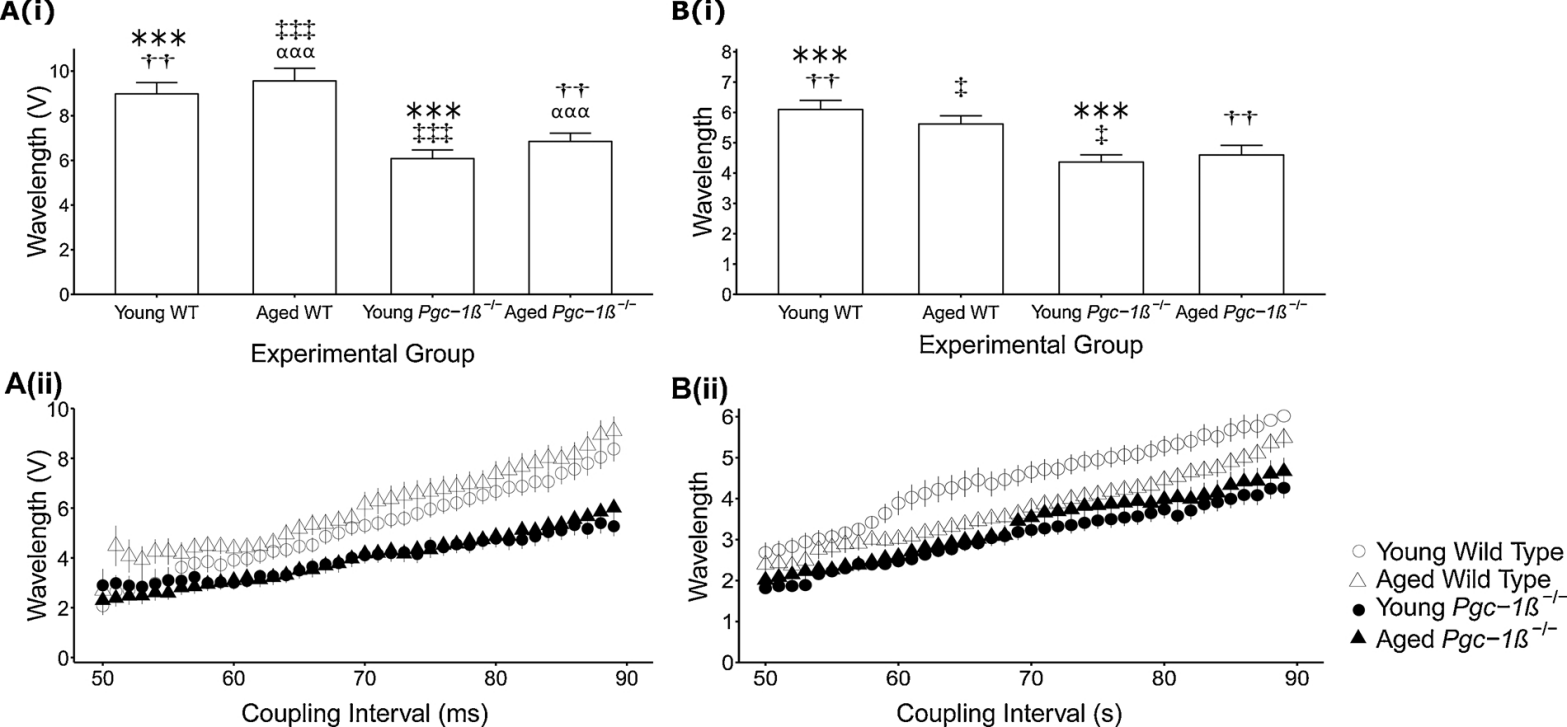
Dependences of AP wavelength assessed using (d*V*/d*t*)_max_ (A(i) and B(i)) and AP latency (A(ii) and B(ii)) through regular 8 Hz pacing (A) and in response to extrasystolic S2 stimulation at different S1-S2 intervals in the PES protocol (B) in young and aged, WT and *Pgc-1β^−/−^* hearts. Wavelength was calculated as the product of the respective conduction parameter (either (d*V*/d*t*)_max_ or AP latency) and the corresponding ERP for that heart. In panels (A(i)) and (A(ii)), symbols denote significant differences between each pair, obtained from post hoc Tukey testing, which was conducted if the ANOVA indicated a significant outcome. Single, double and triple symbols denote p < 0.05, p < 0.01 and p < 0.001 respectively.

Wavelengths made up of AP activation and ERP terms have provided indications of arrhythmic substrate arising from slowed conduction and/or shortened ERP, in earlier experimental analyses of cardiac arrhythmia (Huang, 2017; Matthews et al., 2013; Weiss et al., 2005). Fig. 7 summarises such an analysis for the experimental groups studied during the regular pacing (A) and with the extrasystolic S2 stimuli imposed through the different S1-S2 coupling intervals during PES pacing (B). With (d*V*/d*t*)_max_ as the activation term (Fig. 7A(i) and B(i)), ANOVA demonstrated shorter wavelengths in *Pgc-1β^−/−^* compared to WT ventricles (F = 38.591, p < 0.001) without effects of either age (F = 1.943, p > 0.05) or interactions between age and genotype (F = 0.016, p > 0.05). Post hoc analysis revealed significant differences between each WT group compared to either *Pgc-1β^-/-^* group (young WT *vs.* young *Pgc-1β^-/-^* p < 0.001, young WT *vs.* aged *Pgc-1β^-/-^* p < 0.01, aged WT *vs.* young *Pgc-1β^-/-^* p < 0.001, aged WT *vs.* aged *Pgc-1β^-/-^* p < 0.001). However, there were no significant differences between young or aged groups of the same genotype. This thus accounts for the more marked arrhythmic substrate in both young and aged *Pgc-1β^−/−^*.

With 1/(AP latency) as activation term, representing wavelength by the ratio ERP/(AP latency) thereby including tissue conductivity changes produced by fibrotic change (Fig. 7A(ii) and B(ii)), ANOVA similarly associated *Pgc-1β* ablation with shortened wavelengths compared to WT ventricles (F = 22.766, p < 0.001) with no effect of age (F = 0.36, p > 0.05) or interaction between age and genotype (F = 1.872, p > 0.05). However, the post hoc testing revealed longer wavelengths in young WT than either young *Pgc-1β^−/−^* (p < 0.001) or aged *Pgc-1β^-/-^* (p < 0.01) ventricles. However, wavelengths in aged WT, although higher than in young *Pgc-1β^−/−^* ventricles (p < 0.05), were similar to those shown by aged *Pgc-1β^−/−^* ventricles (p > 0.05). Similarly, dependences of wavelength on S1-S2 coupling intervals showed greater wavelengths in young WT ventricles, shortened values in *Pgc-1β^-/-^* and intermediate measures in aged WT ventricles. Both these findings parallel further findings in Table 1 showing that aged *Pgc-1β^-/-^* hearts displayed fewer individual episodes of abnormal rhythms compared to young *Pgc-1β^-/-^* hearts, and a similar number to the aged WT group.

## 4 Discussion

Mitochondrial dysfunction occurs in age-related conditions including obesity, metabolic syndrome, diabetes mellitus and heart failure and correlates with age related ventricular arrhythmic risk (Adabag et al., 2015; Hookana et al., 2011; Kucharska-Newton et al., 2010). It progresses with age through accumulation of mitochondrial genomic mutations and impaired autophagy (Michikawa et al., 1999; Pyo et al., 2013). It is known to alter cardiomyocyte excitability whether through dysregulated ROS production or interrupted ATP supply (Brown and O’Rourke, 2010; Fischbach et al., 2004; Liu et al., 2010; Sovari et al., 2013; Terentyev et al., 2008; Wang et al., 2004). However, its arrhythmic consequences, particularly under circumstances of chronic mitochondrial impairment, and at the whole organ level, are not well characterized.

Intact Langendorff-perfused murine *Pgc-1β^−/−^* hearts provide suitable models to investigate electrophysiological alterations secondary to metabolic impairment (see Introduction: (Gurung et al., 2011; Lelliott et al., 2006)). Previous *biochemical studies* examining *Pgc-1β* ablation, confirmed regulatory effects upon mitochondrial mass and function, including fatty acid β–oxidation, the tricarboxylic acid cycle and electron transport, as well as levels of pro-inflammatory lipids such as lysophosphatidylcholine (Arany et al., 2005; Gurung et al., 2011). *Cellular studies* reported abnormal diastolic Ca^2+^ transients, negatively shifted Ca^2+^ current inactivation properties and increased inwardly and outwardly rectifying K^+^ currents leading to oscillatory resting potentials, action potentials (APs) with early and delayed after-depolarisations, and burst firing with sustained current injection in isolated *Pgc-1β-/-* ventricular myocytes.

However, few electrophysiological studies have gone on to characterise arrhythmic phenotypes, particularly underlying arrhythmic substrate in *intact Pgc-1β^−/−^* hearts. Preliminary reports described APD alternans and increased frequencies of ventricular tachycardia (VT) (Gurung et al., 2011; Lelliott et al., 2006). They also reported pro-arrhythmic and alternans phenotypes with progressively incremented steady-state pacing rates particularly in aged *Pgc-1β^−/−^* hearts. *Pgc-1β^−/−^* hearts additionally showed reduced maximum action potential (AP) upstroke rates, (dV/dt)_max_ and increased AP conduction latencies (Ahmad et al., 2017).

Sustained arrhythmia likely depends upon interactions between contributions from *triggering events* and *arrhythmic substrate* (Huang, 2017). The present experiments first explored for the presence or absence of such arrhythmic substrate using simultaneous intracellular recordings from left ventricular epicardium following cardiomyocyte activity and volume conductor electrocardiography surveying whole heart activity in modified Langendorff preparations. The presence and extent of arrhythmic substrate was assessed by comparing cellular electrical properties during regular pacing and PES protocols. No arrhythmias occurred with regular baseline pacing consistent with the mild phenotype in unstressed *Pgc-1β^−/−^* hearts. However, PES elicited arrhythmic phenomena preferentially in *Pgc-1β^−/−^* hearts. Here, although greatest incidences were in young *Pgc-1β^−/−^* hearts, aged *Pgc-1β^−/−^* hearts showed more sustained arrhythmic episodes and the greatest proportions of time spent in arrhythmia. WT hearts contrastingly showed few arrhythmias; their higher incidences in aged than young WT hearts paralleled clinical patterns and impacts of age on arrhythmic risk.

Secondly, electrophysiological characterizations of the arrhythmic substrate demonstrated in *Pgc-1β^−/−^* hearts implicated abnormalities in AP initiation and conduction. Close estimates of such activation parameters, (d*V*/d*t*)_max_ and AP latency, and their comparison with AP recovery parameters APD_90_ and ERP, were facilitated by using intracellular as opposed to monophasic AP recordings adopted on previous occasions. Both young and aged *Pgc-1β^−/−^* showed reduced (d*V*/d*t*)_max_ values compared to WT hearts during regular pacing. (d*V*/d*t*)_max_ values were also segregated between genotypes in APs following S2 stimuli during PES protocols. Furthermore, (d*V*/d*t*)_max_ values converged at the shortest intervals that represented the greatest electrophysiological stress. Finally, *Pgc-1β^−/−^* hearts displayed compromised (d*V*/d*t*)_max_ even at modest levels of stress, underpinning their greater vulnerability to arrhythmia through longer periods within the protocol.

Thirdly, *Pgc-1β^−/−^* hearts also showed slow AP conduction reflected in AP latency measurements. However, these increased with ageing both in WT hearts, and to even greater degree in *Pgc-1β^-/-^* hearts. This suggested dependences of AP latency upon genotype and age differing from those found for (d*V*/d*t*)_max_. The latter finding prompted comparisons of AP latency - (d*V*/d*t*)_max_ plots segregating these variables, between young and aged, WT and *Pgc-1β^−/−^* hearts. Latencies of APs elicited by the extrasystolic S2 stimuli increased with falling (d*V*/d*t*)_max_ reflecting established relationships between Na^+^ currents, (d*V*/d*t*)_max_ and conduction velocity (Hunter et al., 1975). However, aged WT hearts showed consistently greater AP latencies at any given (d*V*/d*t*)_max_ than young WT hearts. In contrast, both young and aged *Pgc-1β^-/-^* hearts gave similar functions, matching those from aged WT hearts.

Fourthly, the latter segregation matched histological findings of increased ventricular fibrotic change independently attributable to effects of age and genotype. Aged WT and *Pgc-1β^−/−^* showed greater fibrotic change than corresponding young WT and *Pgc-1β^−/−^* hearts. Furthermore, the greater fibrosis in *Pgc-1β^−/−^* hearts resulted in comparable levels of fibrosis in *young Pgc-1β^-/-^* and *aged* WT hearts, precisely matching the segregation in the AP latency - (d*V*/d*t*)_max_ plots.

Fifthly, no differences in APD_90_ were observed between groups although both young and aged *Pgc-1β^−/−^* hearts showed shorter effective refractory periods (ERPs) than WT, particularly aged WT hearts. Although ERP shortening is often accompanied by corresponding reductions in APD (Lee et al., 1992), this need not be the case in all circumstances and indeed discordant alterations in APD and ERP have been reported previously (Martin et al., 2011). At all events, this ERP shortening would be consistent with increased likelihoods of re-entry in the *Pgc-1β^-/-^* hearts.

Finally, these changes combined into alterations in electrophysiological characteristics matching the observed differences in arrhythmic substrate. AP wavelengths were derived from AP activation ((d*V*/d*t*)_max_ or 1/(AP latency)), and recovery (ERP) terms. The resulting values paralleled the observed relative incidences of arrhythmic substrate in the different groups of young and aged, *Pgc-1β^−/−^* and WT hearts. Thus, use of (d*V*/d*t*)_max_, incorporating Na^+^ current-mediated AP myocyte depolarisation, and 1/(AP latency), further incorporating cardiomyocyte coupling affected by fibrosis both gave reduced wavelength terms in both young and aged *Pgc-1β^−/−^* hearts, recapitulating their relative arrhythmic incidences in Table 1. Post hoc testing of results using 1/(AP latency) went on to suggest higher wavelengths in young WT hearts, and significantly shortened values in the other groups including the aged WT hearts. In post hoc analysis of wavelengths derived from AP latencies, aged WT hearts showed significantly higher values than young *Pgc-1β^−/−^* hearts, accounting for the observed differences in arrhythmic substrate.

Each of the *individual* findings observed here recapitulate specific observations from previous studies characterising murine arrhythmic models with specific *monogenic* ion channel modifications. These first implicated *reduced (dV/dt)_max_*, attributed to primary Nav1.5 insufficiency, in pro-arrhythmic effects in *Scn5a*^+/−^ hearts through reduced AP conduction (Martin et al., 2012), in contrast to the compromised AP recovery in differing, *Scn5a*^+/Δkpq^ and *Kcne5*^-/-^ pro-arrhythmic systems (Thomas et al., 2008, 2007). The reduced (d*V*/d*t*)_max_ observed in cardiomyocytes during sharp electrode recordings in *intact tissue* contrasts with normal or even enhanced Na^+^ currents in *isolated patch-clamped Pgc-1β^-/-^* cardiomyocytes. However, whereas the present findings were made under conditions of unperturbed intracellular homeostasis, the latter were necessarily involved Ca^2+^ chelation by intrapipette BAPTA (Gurung et al., 2011)

This comparison suggests an *in vivo* mechanism of action involving acute effects of the altered Ca^2+^ homeostasis observed in *Pgc-1β^−/−^* hearts upon membrane excitability, potentially arising from interactions between cytosolic Ca^2+^ and Nav1.5. Thus, direct Ca^2+^ - Na_V_1.5 binding can occur at an EF hand motif near the Na_V_1.5 carboxy-terminal (Wingo et al., 2004). In addition, indirect Ca^2+^ binding could involve an additional ‘IQ’ domain Ca^2+^/CaM binding site in the Na_V_1.5 C-terminal region (Grandi and Herren, 2014; Mori et al., 2000; Wagner et al., 2011). These findings then add a metabolic example of intracellular Ca^2+^ homeostasis affecting arrhythmic substrate through altered AP propagation. Previous reports have described reversible *I*_Na_ and (d*V*/d*t*)_max_ reductions in patch clamped cardiomyocytes following intracellular [Ca^2+^] elevations (Casini et al., 2009). *I*_Na_ reduction similarly follows both other situations involving metabolic stress (Liu et al., 2010, 2009) and cytosolic [Ca^2+^] elevation in intact hearts. They also accompanied the *slowed AP conduction* in intact hearts following caffeine challenge (Zhang et al., 2009) or *RyR2*-P2328S modification, both associated with diastolic Ca^2+^ release (Glukhov et al., 2013; Huang, 2017; King et al., 2013b; Li et al., 2014; Ning et al., 2016; Salvage et al., 2015; Zhang et al., 2013). Such diastolic Ca^2+^ transients similarly occur in *Pgc-1β^-/-^* myocytes (Gurung et al., 2011).

Secondly, altered cardiomyocyte coupling further limiting AP propagation, with *increased AP latency*, occurs with connexin Cx40 or Cx43 deficiency (Royer et al., 2005; Simon et al., 1998; Verheule and Kaese, 2013), impaired gap junction function due to elevated intracellular [Ca^2+^] (De Mello, 1983), altered connexin phosphorylation (Sovari et al., 2013) and tissue fibrotic changes associated with Nav1.5 haploinsufficiency (Jeevaratnam et al., 2012, 2011). These potentially alter intracellular electrical resistances coupling adjacent cardiomyocytes (Xie et al., 2009) as well as cardiomyocyte-fibroblast fusion (Davies et al., 2014; Mahoney et al., 2016; Xie et al., 2009) the latter increasing effective membrane capacitances (Chilton et al., 2007; van Veen et al., 2005).

Thirdly, independent evidence similarly exists for *fibrotic change* associated with *age* in other experimental (Eghbali et al., 1989; Jeevaratnam et al., 2012; Orlandi et al., 2004) and clinical situations (Gazoti Debessa et al., 2001); these in turn affect AP conduction (Jeevaratnam et al., 2011; King et al., 2013a). Previous reports also implicate *mitochondrial ROS in fibrotic change* (Brown and O’Rourke, 2010; Liu et al., 2010; Terentyev et al., 2008; Wang et al., 2004). Such cardiac fibrosis was reduced by catalase overexpression (Dai et al., 2009) and accentuated by mitochondrial sirtuin SIRT3 deficiency, in transgenic mice (Hafner et al., 2010). Oxidative stress similarly enhances TGF-β activity (Barcellos-Hoff and Dix, 1996; Sullivan et al., 2008) that has been postulated to promote age-related myocardial fibrosis (Brooks and Conrad, 2000; Davies et al., 2014; Rosenkranz et al., 2002).

## 5 Conclusion

Together our work in cardiomyocytes and overall electrophysiological activity in intact hearts thus extends initial studies on the *Pgc-1β^−/−^* model at the cellular level to obtain electrophysiological evidence for increased arrhythmic substrate under conditions of chronic mitochondrial deficiency. We attribute this predominantly to a reduction in measures of AP conduction, as reflected in (d*V*/d*t*)_max_ and AP latency, and demonstrate this is accompanied by increased fibrotic change with age in *Pgc-1β^-/-^* hearts.

## Conflicts of interest

None declared.
